# Editorial: Bypassing the Biological Barriers by Means of Biocompatible Drug Delivery Systems

**DOI:** 10.3389/fphar.2021.801383

**Published:** 2021-11-24

**Authors:** Vibhudutta Awasthi, Stefania Bulotta, Donato Cosco

**Affiliations:** ^1^ Department of Pharmaceutical Sciences, The University of Oklahoma Health Sciences Center, Oklahoma City, OK, United States; ^2^ Department of Health Sciences, University “Magna Græcia” of Catanzaro, Campus Universitario “S. Venuta”, Catanzaro, Italy

**Keywords:** biobarriers, polymeric nanoparticles, drug delivery, BBB, skin

Passage of a drug across a biomembrane, such as a cell bilayer, vascular endothelium and bowel epithelium, the skin, the blood-brain barrier, is influenced by its physicochemical properties and characteristics of the biomembrane (Shinoda, 2016; Di Meo et al., 2016). Biomembranes contain cellular material in a closed space and provide a dynamic interface for interaction with the extracellular milieu. However, from a pharmaceutical perspective, biomembranes can also act as a barrier to drug delivery. Knowledge of these influences can provide us with a capability to localize drugs in specific body compartments by modifying the delivery systems, altering the route of administration, and incorporating the compartment-specific targeting moieties. With the goal of promoting drug permeation across a biomembrane, these techniques and approaches essentially hinge on the modulation of the interaction between the drug and the bilayer. This special issue is a collection of articles describing the state of the art pertaining to the use of biocompatible polymeric nanoparticles as drug carriers of bioactive compounds. The research articles discuss the *in vivo* fate of polymeric nanosystems in relation to the unique characteristics of the tumor microenvironment. A couple of other articles discuss the delivery of drugs through the cutaneous route.

Torres et al. reviewed the nature of biobarriers and strategies to overcome them for the delivery of anticancer drugs. Specifically, the authors described nanocarriers composed of stimuli-responsive and self-assembling amphiphilic block copolymers to modulate the release of the payload. Exemplified by polylysine-cholate/PEG matrix to carry doxorubicin and deliver by pH stimuli (Zhu et al., 2012), such polymers are biocompatible and their compositions are tunable to match desired circulation persistence and target-oriented surface functionalization. Drug delivery by magnetic stimuli is another approach investigated by Li et al. The researchers employed an external magnetic field to induce localization of zolendronate-PLGA nanoparticles carrying superparamagnetic iron oxide in joints of a mouse model of osteolysis. They loaded these nanoparticles with curcumin to create dual-targeted nanoparticles and showed inhibition of NF-κB signaling and enhanced efficacy against polyethylene-induced osteolysis. Extending the application of stimuli-driven delivery of drugs, Oddone et al. engaged reactive oxygen species (ROS)-responsive mPEG-thioketal-melphalan prodrug for the potential treatment of glioblastoma. Although no *in vivo* efficacy studies were conducted, the investigators characterized the delivery system and tested it in cell culture. Thioketal linkage is sensitive to ROS and cleavage of thioketal bond releases melphalan from the micellar assembly of the drug-polymer conjugate. Because ROS levels are higher in cancer cells than in normal cells, such systems are expected to show selective cytotoxicity against cancer cells. In addition, the use of concomitant radiotherapy can enhance the anticancer effect of mPEG-thioketal-melphalan prodrug by increasing ROS levels.

For topical drug delivery, the skin acts as a biological barrier. It is the largest organ of our body and its various strata limit the permeation of substances. Here, Tumpara et al. proposed a simple topical delivery to replace traditionally employed intravenous infusion of α1-antitrypsin (AAT) protein (molecular weight ∼52 kDa). They evaluated transepidermal passive diffusion of AAT from buffered solution using 3D epidermis inserts reconstructed from primary human epidermal keratinocytes. The researchers reported that AAT was freely diffused across the epidermis layers in a concentration- and time-dependent manner. More importantly, there was no damage to the keratinocyte layers and AAT application reduced the inflammatory markers (IL-1α and IL-18) induced by LPS treatment. Given the simplicity of the approach, this cutaneous delivery of AAT warrants *in vivo* testing, as it will provide a new administration route to treat patients with inherited AAT deficiency.

One of the challenges in skin delivery is the real-time monitoring of payload after application. Snoswell et al. proposed a novel method to monitor cutaneous drug delivery. This method is based on motion capture and shows promise both as a training tool and as a means to assist in the development of skin drug delivery technologies. The investigators demonstrated the use of a “pen-type” applicator to capture the motion of Na-fluorescein-loaded elongated microparticles within *ex vivo* porcine skin samples; the permeation of microparticles was evaluated as a function of fluorescence, azimuth angle of application, frequency, and time. Since this is a non-invasive technique, it can be translated in humans to monitor drug delivery and detect mechanical differences among individuals, especially when significant variations in pharmacological outcomes or clinical efficacy are expected.

In summary, the articles in this special issue provide an overview of challenges posed by biological membranes and a few strategies to overcome them. The approaches described herein have in common the requisites to avoid the permanent destabilization of biobarriers to improve drug permeation. Encapsulation or complexation of drugs within polymeric micro/nanocarriers is one of many strategies to achieve this goal, mainly for parenteral administration (Maghrebi et al., 2019; Yari et al., 2020; Voci et al., 2021); a few such nanoformulations are now approved for clinical application or are under investigation (Mura et al., 2019; Jena et al., 2020; Sofias et al., 2021). Techniques and innovative formulations have also been developed to increase the skin delivery of the bioactives (Aich et al., 2021; Amani et al., 2021; Kaur and Kesharwani. 2021; Roberts et al., 2021). Although clinical development of drug and formulation products of nanomedicine class is still in its infancy (He et al., 2019), the guest editors opine that the advances in new biocompatible polymeric compositions and innovative non-invasive monitoring techniques will hasten the clinical translation of this research.
